# Risk factors for febrile respiratory illness and mono-viral infections in a semi-closed military environment: a case-control study

**DOI:** 10.1186/s12879-015-1024-7

**Published:** 2015-07-25

**Authors:** Junxiong Pang, Jing Jin, Jin Phang Loh, Boon Huan Tan, Wee Hong Victor Koh, Sock Hoon Ng, Zheng Jie Marc Ho, Qiuhan Gao, Alex R Cook, Li Yang Hsu, Vernon J Lee, Mark I Cheng Chen

**Affiliations:** Centre for Infectious Disease Epidemiology and Research, Saw Swee Hock School of Public Health, National University of Singapore, Singapore, Singapore; Communicable Disease Centre, Institute of Infectious Diseases and Epidemiology, Tan Tock Seng Hospital, Singapore, Singapore; Defence Medical and Environmental Research Institute, Singapore, Singapore; Biodefence Centre, Ministry of Defence, Singapore, Singapore; Yale-NUS College, National University of Singapore, Singapore, Singapore; Program in Health Services and Systems Research, Duke-NUS Graduate Medical School, Singapore, Singapore; Department of Statistics and Applied Probability, National University of Singapore, Singapore, Singapore; Department of Medicine, National University of Singapore, Singapore, Singapore

**Keywords:** Febrile respiratory infections, Risk factors, Military, Influenza A(H1N1)pdm09, Influenza B, Coxsackie/Echovirus, Adenovirus, Rhinovirus

## Abstract

**Background:**

Febrile respiratory illness (FRI) results in substantial burden in semi-closed environments. Tackling risk factors may reduce transmission and infection. However, risk factors involved in one setting may not be generalizable in all settings due to differences in climate, residential environment, population genetic and cultural backgrounds. This study aims to identify risk factors of FRI and mono-viral infections in a tropical military environment.

**Methods:**

From year 2009 to 2012, military personnel with temperature ≥37.5 °C, cough and/or sore throat, and personnel with no fever or no respiratory symptoms were recruited as cases and controls, respectively. Subjects provided nasal wash specimens and answered a standardized questionnaire. Resplex assays were used to determine the viral etiologies. Descriptive, univariate and multivariate analyses of the variables were performed using appropriate descriptive tests and logistic regression modelling, respectively, with R program.

**Results:**

A total of 7,743 FRI cases and 1,247 non-FRI study controls were recruited. Increasing age [adjusted odds ratio (AOR) = 1.03; 95 % confidence interval (CI) = 1.01-1.05], recruit camp (AOR = 4.67; 95 % CI = 3.99-5.46) and smoker (AOR = 1.31; 95 % CI = 1.13-1.52) were independent risk factors of FRI. Malay ethnicity was positively associated with influenza A(H1N1)pdm09 (AOR = 1.50; 95 % CI = 1.04-2.15) and coxsackie/echovirus (AOR = 1.67; 95 % CI = 1.19-2.36) mono-infection. Significant contact risk factors were stay-out personnel with ill household member (AOR = 4.96; 95 % CI = 3.39-7.24), and stay-in personnel with ill bunkmate and household member (AOR = 3.55; 95 % CI = 2.57-4.91). Staying in camp with none ill in bunk and at home was a protective factor against FRI (AOR = 0.80; 95 % CI = 0.64-0.99). These contact risk factors were similarly observed for the five most common viruses detected, namely adenovirus, rhinoviruses, influenza A and B, and coxsackie/echovirus.

**Conclusion:**

Increasing age, smoker, recruit-camp, stay-out personnel with ill household members and stay-in personnel with ill bunkmates were independent risk factors of FRI in a semi-closed military environment. Early identification and isolation of ill personnel from their bunk may be effective to prevent and reduce transmission and disease burden.

## Background

Febrile respiratory illness (FRI) results in substantial disease burden in semi-closed environments such as in the households [1] and militaries [2–4]. FRI is most commonly caused by viral infections, as observed in military respiratory surveillance programmes in Finland [5], United Kingdom [4, 6–9], Netherlands [10], France [11, 12], South Korea [13–15], West Africa [16], Taiwan [17], China [18], Singapore [19–24], and the United States [3, 25–29].

Identifying risk factors of infection may provide guidance on policies and strategies for the prevention and control of FRI. Previous documented risk factors of FRI in other countries included body mass index equal or greater than 25 kg/m^2^, previous respiratory tract infections [30], overcrowding and closed units [29, 31–33], presence of sand and dust storms, extreme temperature changes [34, 35], smoking [36], female, Navy service, poor latrine facilities, increasing age and higher rank [37]. However, these risk factors may not be generalizable to different environments, and may differ between specific predominant aetiological agents.

The predominant viruses reported in the Singapore Armed Forces (SAF) comprised adenovirus, rhinoviruses, influenza A and B, and coxsackie/echovirus between 2009 and 2012 [21]. Adenovirus-associated respiratory disease, outbreaks and death have been reported in several countries amongst military recruits [10, 13, 14, 18, 19, 21, 27, 38–43]. Males and close contact with a person with respiratory symptoms within 10 days before their own onset of illness were associated with adenovirus infection, but sleeping adjacent to someone ill with respiratory symptoms did not present higher risk to infection [39]. Influenza A and B viruses have also resulted in much morbidity in outbreaks, particularly influenza A(H1N1)pdm09 virus infection [11, 12, 17, 20, 21, 26, 44]. Some of the risk factors proposed were crowded living quarters defined as more than three personnel and age group less than 40 year old [45], asthma and obesity [46], age group less than 30 years old and the high proportion of military who had being seroconverted [47]. Human rhinoviruses are known to cause common cold as well as more complicated respiratory infections [9, 48–52]. All known human rhinoviruses have been reported to be present in military recruits during respiratory infection [53]. Association of rhinovirus with lower respiratory tract infections is well documented [54]. Viral interference has also been proposed between rhinovirus and adenovirus infection [38]. Stress factor due to adaptation to new and different surroundings for military recruits was also proposed as risk factor for rhinovirus infection [48]. In this study, we investigate the risk factors associated with FRI and the predominant viral aetiologies of FRI in a semi-closed military environment of the SAF.

## Methods

### Study setting and data collection

The SAF started a sentinel respiratory disease surveillance program in four major camps (including a recruit training camp) in May 2009 [21, 22] to track febrile respiratory illness (FRI) cases defined as patients with temperature ≥37.5 °C with cough or sore throat. Patients visiting primary healthcare clinics in the camps between May 2009 and October 2012 during regular consultation hours who met the FRI criteria were recruited. The sentinel respiratory disease surveillance program includes the written informed consent obtained by healthcare workers, a questionnaire, clinical specimens collection and a clinical examination of the participants. Repeat consultations were excluded if the healthcare worker determined that the patient had not recovered from the first episode of illness. We also obtained samples from controls (those without respiratory symptoms or acute infections), who were recruited from the same medical center during the same week as the recruitment of cases with about 5 to 10 controls per week. This is to ensure that both cases and controls had similar health-seeking behaviour, and similar chance of exposure to a particular respiratory pathogen circulating in the same environment around the same period of the year to minimize potential misclassification bias. Moreover, the controls were not matched or restricted by barrack, sex, age or symptom-onset. This is because of the fact that the aim of the study is to evaluate most of these variables as potential risk factors of FRI. Informed consent, the baseline questionnaire, and clinical specimens were also obtained from these controls.

The questionnaire covers demographics, co-morbidities, vaccination status, stay-in camp status and contact details of ill member in bunk (for stay-in personnel) and at home (for both stay-in and stay-out personnel). Stay-in personnel stay in camp on weekdays and stay outside camp only on weekends, and hence, have household members and bunkmates as their key contacts. Stay-out personnel do not stay inside camp on weekdays and have to travel in and out of camp on weekdays to work. These stay-out personnel hence only have household members but no bunkmates as key contacts.

**Fig. 1:**
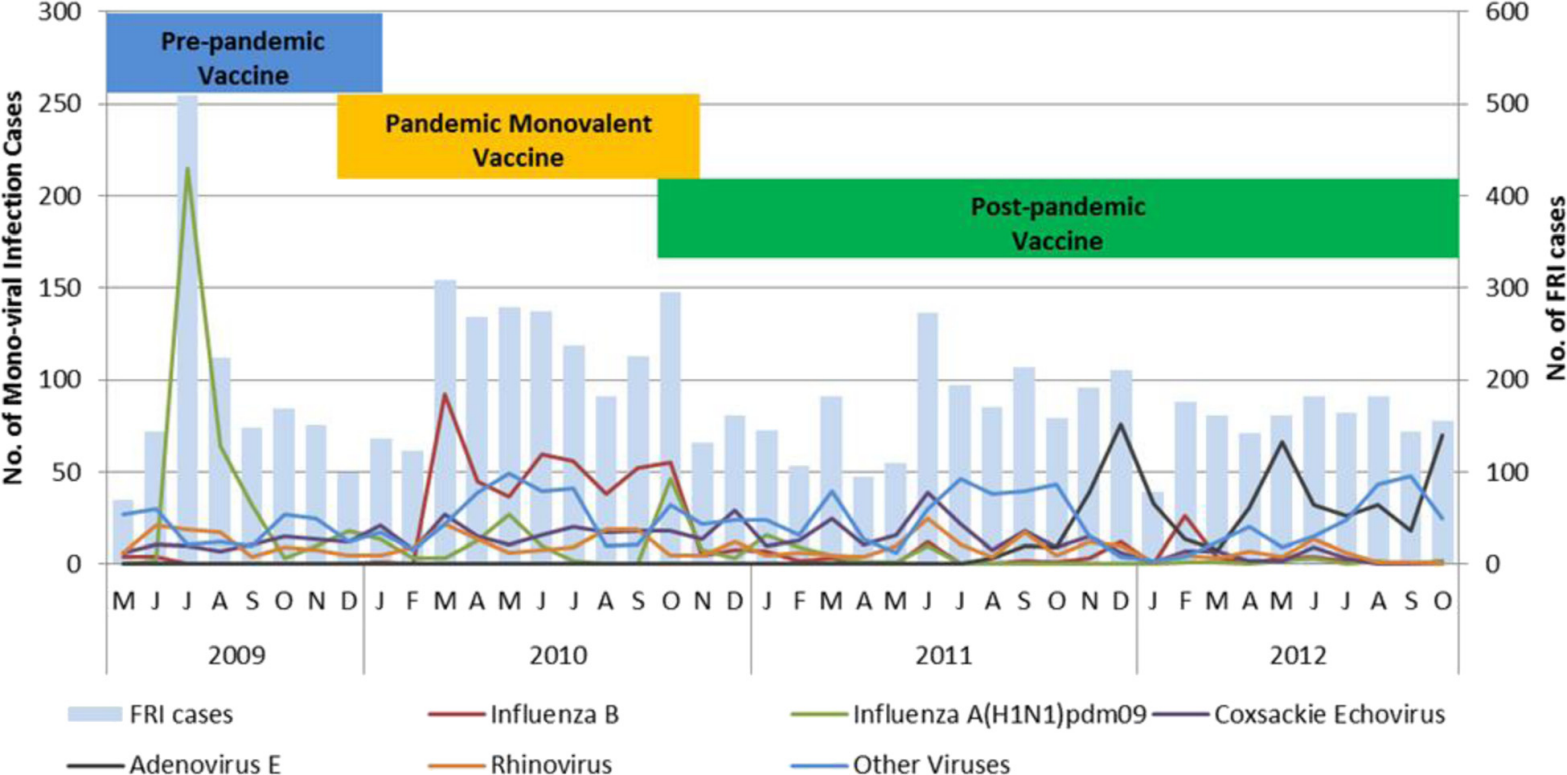
Monthly cases of FRI and the five most common mono-viral infections from year 2009 to 2012 with the different phases of vaccine programme implementation

Before the influenza A(H1N1)pdm09 pandemic in 2009, trivalent inactivated seasonal influenza vaccine (pre-pdm TIV) was in use. Then, the pandemic monovalent influenza A(H1N1)pdm09 vaccine [pdm-A(H1N1)V] was first introduced to SAF and administered to all recruits only from December 2009. This was superseded by the new trivalent influenza vaccine (post-pdm TIV) which included the influenza A(H1N1)pdm09 strain, first introduced to SAF in October 2010, and routinely administered to all recruits in December 2010, and then all other military personnel in November 2011 (Fig. 1).

### Laboratory methods

Nasal washes from both side of the nose were taken by certified medical staff and sent to the laboratory for etiological testing within 24 h. Detailed laboratory methods have been described in a previous study [21, 55]. Briefly, we used a multiplex PCR panel which included 18 different respiratory viruses. They are as following- Adenovirus E, Influenza A(H3N2), Rhinovirus, Coxsackie/Echovirus, Influenza B, influenza A(H1N1)pdm09, Enterovirus (EV), human metapneumovirus (hMPV), Parainfluenza 1 (hPIV-1), hPIV-2, hPIV-3 and hPIV-4, Coronavirus OC43 (CoV-OC43), CoV-NL63, CoV-229E, CoV-HKU1, respiratory syncytial virus A (RSV-A) and RSV-B and Bocavirus (BV). Additional singleplex PCR assays were then performed to determine the influenza subtype. Total nucleic acids were extracted from each clinical specimen using the DNA minikit (Qiagen, Inc, Valencia, CA, USA) according to the manufacturer’s instructions. A total of 20 μl of DNA extract were tested with Resplex I and II (version 2.0, Qiagen, Inc., Valencia, CA, USA) for the presence of respiratory micro-organisms on the LiquiChip 200 Workstation, according to the manufacturer’s instructions. Specimens that were Resplex II positive for FLU-A were further subtyped with real-time PCR for H1 or H3, or for pH1N1. Briefly, 5 μl of total genetic extracts were tested using an in-house developed assay based on the one-step SuperscriptIII/Platinum Taq kit (Invitrogen, Carlsbad, CA, USA) following the manufacturer’s instructions on either the LightCycler machine from Roche or the Applied Biosystems real-time PCR machine (7500).

### Statistical analysis

We compared variables of all FRI subjects, and subsets of subjects with mono-viral infection (MVI) for the five most common viral pathogens (case groups) against the non-FRI patients without viral infection detected from the panel (control group). Specifically, the five most common viruses were influenza B, influenza A (H1N1) pdm2009, coxsackie/echovirus, adenovirus E and rhinovirus. Univariate logistic regression was conducted to identify statistically significant variables of interest. Selected variables with high co-linearity were dropped, but all other variables significant at *p* < 0.05 were then included in a multivariable logistic regression model to determine the independent factors. The best model was determined using backward stepwise regression method. Power calculation showed at least 84 % power to detect a true positive association with effect size of 1.4 with 30 % of the controls having the exposure of interest as cases. All tests were conducted at the 5 % level of significance. We report odds ratio (OR) and corresponding 95 % confidence intervals (CI) where applicable. All statistical analyses were performed using an open source statistical software R 3.0.1 (R Core Development Team).

### Ethics

Written informed consent was obtained from the study participants. This study was reviewed and approved by the Singapore military’s Joint Medical Committee for Research, and the National University of Singapore’s ethics review committee.

## Results

### Demographic characteristics and vaccination history of FRI cases and non-FRI controls

A total of 7,743 FRI cases were recruited. Of these, there were 3,422 FRI cases (44.2 %) with mono-viral infection (MVI). Of the 3,422 MVI cases, the five most common MVI were due to influenza B with 541 cases (15.8 %), influenza A (H1N1)pdm09 with 526 cases (15.4 %), coxsackie/echovirus with 523 cases (15.3 %), adenovirus E with 467 cases (13.6 %), and rhinovirus with 378 cases (11 %); the number of cases observed in each month from May 2009 to October 2012 is shown in Fig. 1. Of the 1,365 non-FRI subjects recruited, 1,247 subjects (91.4 %) were confirmed to be negative for the whole panel of respiratory pathogens tested, and these served as the study controls in all subsequent analysis.

The mean age of FRI cases was 20.8 (±3.12) as compared to 21.0 (±2.62) years old for controls (*P* < 0.001; Table 1). A significantly higher proportion of cases (71.8 %) came from the recruit camp as compared to the controls (33.7 %; *P* < 0.001). The proportion of FRI cases who had pre-pdm TIV and post-pdm TIV were significantly lower and higher than the study controls, respectively (2.3 % vs 3.9 %, *p* = 0.002, and 31.0 % vs 22.4 %, *p* < 0.001, respectively; Table 1). In addition, there were significant differences in the smoking status among the cases compared to the controls (*p* = 0.028), and there were significantly higher proportion of FRI cases than the study controls (20.6 % vs 17.2 %, *p* = 0.004). In terms of movement history, there was a significantly lower proportion of cases who had travelled to other camp in the last 14 days before clinical presentation than that of study controls (6.1 % vs 14.2 %; *p* < 0.001), and there were significantly higher proportion of FRI cases who stayed in camp compared to the controls (86.9 % vs 81.6 %, *p* < 0.001; Table 1).

**Table 1:** Demographics of FRI cases, and non-FRI study controls

Characteristic	FRI cases (N = 7743)	Percent	Non-FRI study controls (N = 1247)	Percent	*P*-value^a^
Age:					
Mean (SD)	20.8	(3.12)	21.0	(2.62)	<0.001^b^
Range	17-60		18-55		
Gender:					
Male	7723	99.7	1246	99.9	0.354
Ethnic groups					
Chinese	5834	75.4	972	77.9	
Malay	1151	14.9	158	12.7	
Indian	510	6.6	88	7.1	
Others	248	3.2	29	2.3	0.344
Camp					
Non-recruit camp	2186	28.2	827	66.3	<0.001
Recruit camp	5557	71.8	420	33.7	
Pre-pdm TIV:					
Yes	180	2.3	49	3.9	0.002
Post-pdm TIV:					
Yes	2400	31.0	279	22.4	<0.001
Pdm-A(H1N1)V:					
Yes	1335	17.2	222	17.8	0.629
Smoking					
Non-smoker	5388	69.6	877	70.3	
Current-smoker	2094	27.1	346	27.7	
Ex-smoker	257	3.3	24	1.9	0.028
Asthma					
Yes	1598	20.6	215	17.2	0.004
Heart disease					
Yes	83	1.1	15	1.2	0.763
Diabetes					
Yes	8	0.1	2	0.2	0.656
Hypertension					
Yes	45	0.6	6	0.5	0.700
Travelled to community in last 14 days:					
Yes	7670	99.1	1238	99.3	0.548
Travelled to other camp in last 14 days:					
Yes	471	6.1	177	14.2	<0.001
Travelled overseas in the last 14 days:					
Yes	227	2.9	49	3.9	0.057
Camp stay					
Stay-in	6928	89.6	1018	81.6	<0.001

^a^Chi-squared test; ^b^unpaired *t*-test; Pdm- pandemic

### Demographic risk factors of FRI

**Fig. 2:**
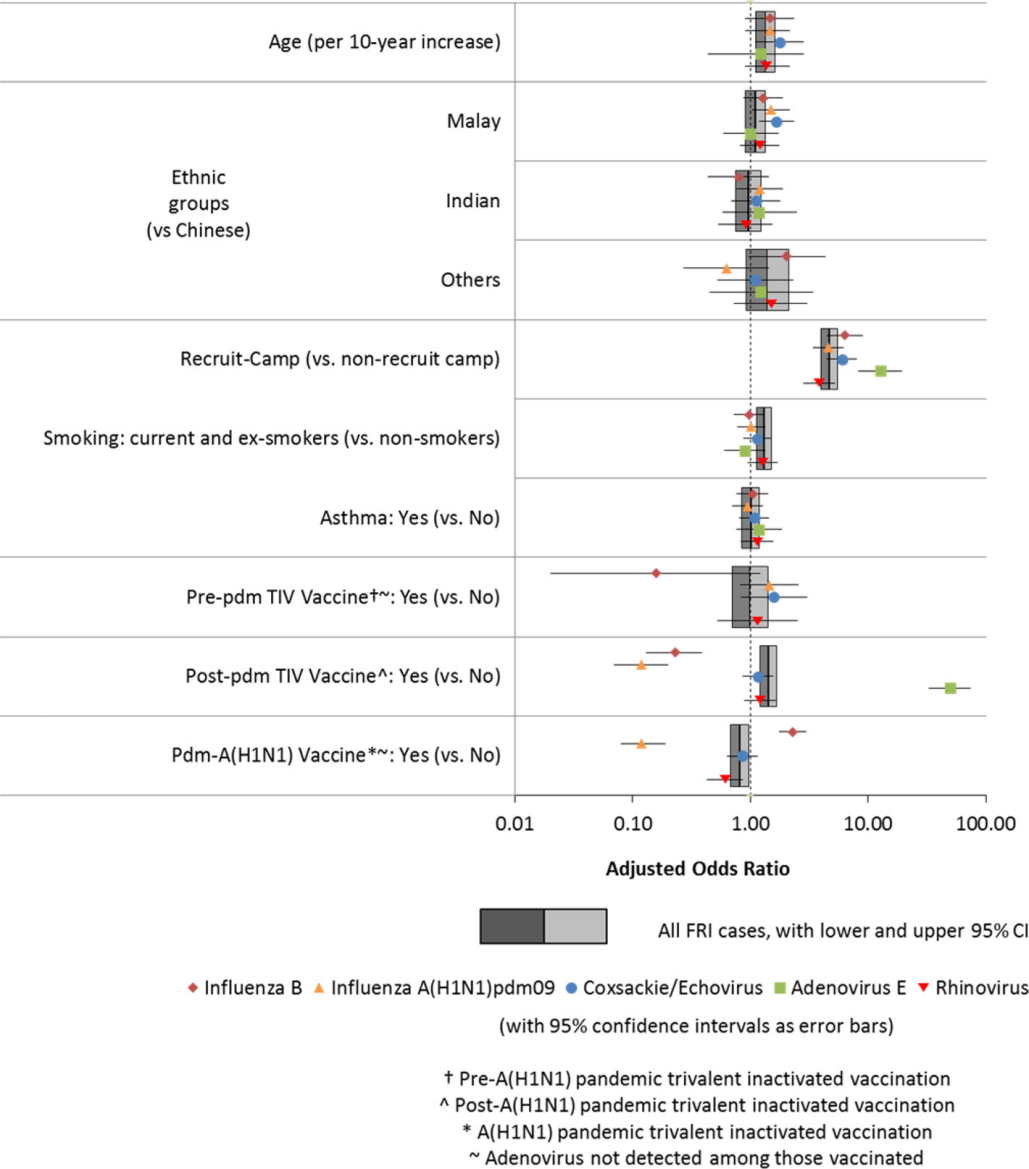
Demographic risk factors for FRI and the five most common mono-viral infections

Increasing age was observed to be an independent risk factor for FRI [adjusted odds ratio (AOR) = 1.03; 95 % confidence interval (CI) = 1.01-1.05; Fig. 2]. In addition, recruit camp (AOR = 4.67; 95 % CI = 3.99-5.46), post-pdm TIV (AOR = 1.42; 95 % CI = 1.21-1.66), and smoker (AOR = 1.31; 95 % CI = 1.13-1.52) were independent risk factors for FRI. Personnel vaccinated with pdm-A(H1N1)V had reduced risk of FRI by 1.23 times (AOR = 0.81; 95 % CI = 0.68-0.97). While pre-pdm TIV reduced the risk of FRI by 2.22 times (COR = 0.58; 95 % CI = 0.42-0.80), it was not independently associated with FRI after adjusting for potential confounding factors (Table 2). Similarly, asthma (COR = 1.25; 95 % = 1.07-1.46) was a potential risk factor of FRI, but it was not independently associated with FRI after adjusting for potential confounding factors (Table 2).

**Table 2:** Risk factors of FRI

Characteristic	Crude OR	95 % CI		Adjusted OR^a^	95 % CI	
Age	**0.97**	**0.96**	**0.99**	**1.03**	**1.01**	**1.05**
Gender: Male	0.31	0.04	2.31			
Ethnic groups						
Chinese	1.00					
Malay	**1.21**	**1.01**	**1.45**	1.10	0.90	1.34
Indian	0.97	0.76	1.22	0.96	0.75	1.24
Others	1.42	0.96	2.11	1.39	0.92	2.11
Camp						
Non-recruit camp	1			1		
Recruit camp	**5.01**	**4.41**	**5.69**	**4.67**	**3.99**	**5.46**
Pre-pdm TIV:						
Yes	**0.58**	**0.42**	**0.80**	0.99	0.70	1.41
Post-pdm TIV:						
Yes	**1.56**	**1.35**	**1.80**	**1.42**	**1.21**	**1.66**
Pdm-A(H1N1)V:						
Yes	0.96	0.82	1.12	**0.81**	**0.68**	**0.97**
Smoking						
Ex-/Current Smoker	1.03	0.91	1.18	**1.31**	**1.13**	**1.52**
Asthma						
Yes	**1.25**	**1.07**	**1.46**	1.01	0.85	1.19
Heart disease						
Yes	0.89	0.51	1.55			
Diabetes						
Yes	1.21	0.51	2.84			
Hypertension						
Yes	0.64	0.14	3.04			
Travelled to community in last 14 days:						
Yes	0.76	0.38	1.53	0.84	0.40	1.77
Travelled to other camp in last 14 days:						
Yes	**0.39**	**0.33**	**0.47**	0.87	0.71	1.06
Travelled overseas in the last 14 days:						
Yes	0.74	0.54	1.01	1.08	0.77	1.52
Camp Stay & ill contacts						
Stay-out & ill household member	**4.59**	**3.16**	**6.67**	**4.96**	**3.39**	**7.24**
Stay-in, none ill in bunk and at home	**1.24**	**1.01**	**1.52**	**0.80**	**0.64**	**0.99**
Stay-in, none ill in bunk & ill household member	**1.63**	**1.14**	**2.33**	1.17	0.80	1.70
Stay-in, ill bunkmate & none ill at home	**4.46**	**3.66**	**5.42**	**1.75**	**1.39**	**2.20**
Stay-in, ill bunkmate and household member	**6.87**	**5.07**	**9.32**	**3.55**	**2.57**	**4.91**

^a^Adjusted for age, ethnic groups, camp, vaccination type, smoking, asthma, travel history, camp stay and ill contacts

Pdm- pandemic

Of the five most common MVI, increasing age was positively associated with coxsackie/echovirus(AOR = 1.06; 95 % CI = 1.01-1.11; Fig. 2), and Malay ethnicity was positively associated with influenza A(H1N1)pdm09 (AOR = 1.50; 95 % CI = 1.04-2.15,) and coxsackie/echovirus (AOR = 1.67; 95 % CI = 1.19-2.36) mono-infection. As compared to personnel in non-recruit camps, those in recruit camps had a higher risk for all the five MVI, with the highest risk for adenovirus E (AOR = 12.70; 95 % CI = 8.31-19.41) and the lowest risk for rhinovirus mono-infection (AOR = 3.83; 95%CI = 2.83-5.19). Personnel with post-pdm TIV had 4.35 and 8.33 times lower risk of influenza B (AOR = 0.23; 95 % CI = 0.13-0.39) and influenza A(H1N1)pdm09 (AOR = 0.12; 95 % CI = 0.07-0.20) mono-infection, respectively. On the contrary, post-pdm TIV was positively associated with adenovirus mono-infection (AOR = 49.51; 95 % CI = 32.91-74.48). Receipt of pdm(H1N1)V was associated with lower risk of influenza-A(H1N1)pdm09 and rhinovirus mono-infection by 8.33 times (AOR = 0.12; 95 % CI = 0.08-0.19) and 1.67 times (AOR = 0.61; 95 % CI = 0.43-0.86), but positively associated with influenza-B mono-infection (AOR = 2.28; 95 % CI = 1.75-2.96).

### Contact risk factors of FRI

**Fig. 3:**
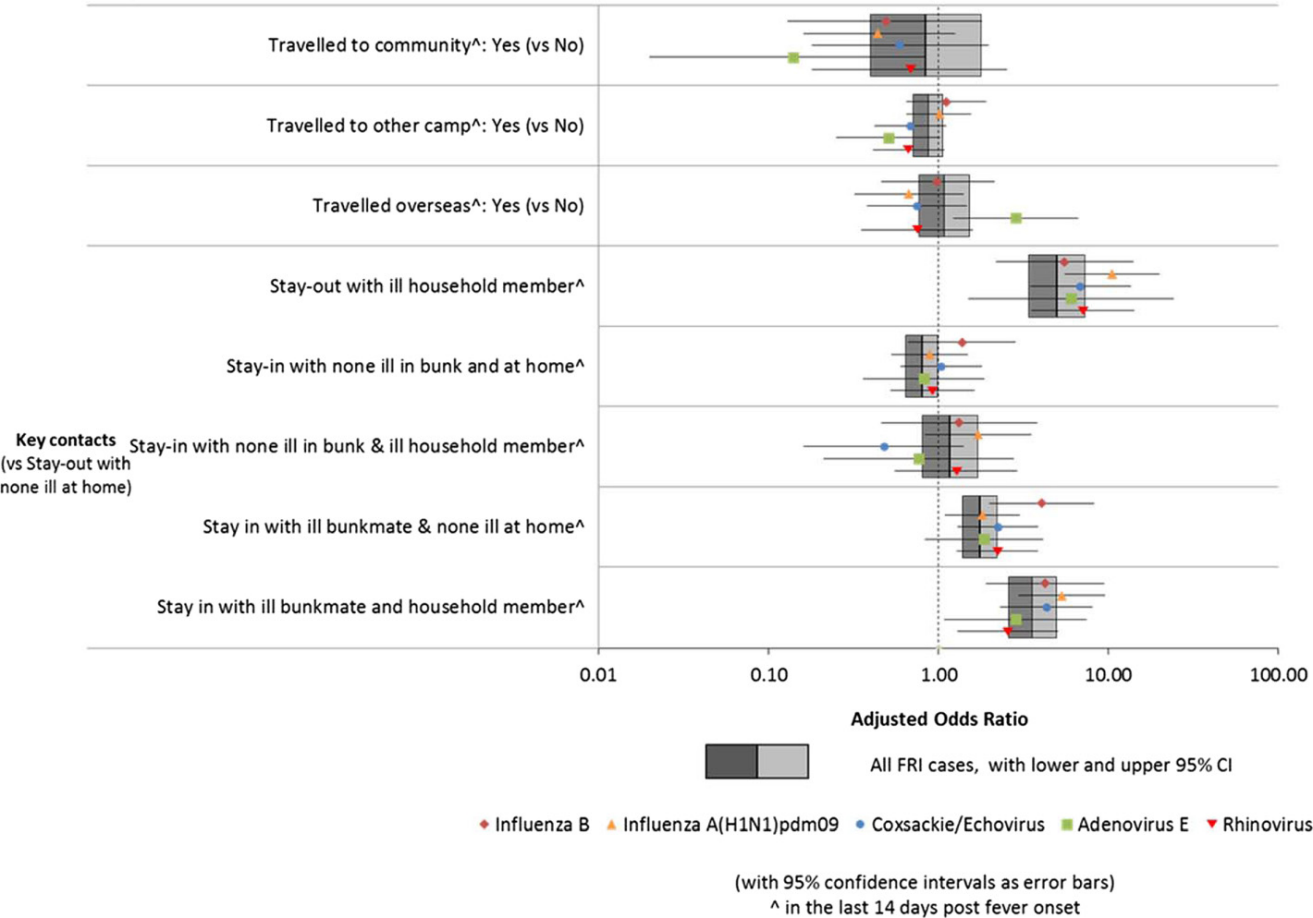
Contact risk factors for FRI and the five most common mono-viral infections

Personnel who travelled to the community in the last 14 days before clinical presentation had a significantly lower risk of adenovirus mono-infection (AOR = 0.14; 95 % CI = 0.02-0.84; Fig. 3) compared to personnel who did not. However, personnel travelling overseas in the last 14 days before clinical presentation had 2.85 times higher risk of adenovirus mono-infection (AOR = 2.85; 95 % CI = 1.22-6.65) compared with personnel who did not travel overseas. Compared to stay-out personnel with no ill household members in the last 14 days before clinical presentation, stay-out personnel with ill household members had 4.99 times higher risk of FRI (AOR = 4.95; 95 % CI = 3.39-7.293). Moreover, compared to stay-out personnel with no ill household members, stay-in personnel who had neither ill bunkmates nor household members had 1.28 times lower risk of FRI (AOR = 0.80; 95 % CI = 0.64-0.99). However, there was a higher risk of FRI for stay-in personnel with an ill member in bunk regardless of whether they had any ill household members (AOR = 3.55; 95 % CI = 2.57-4.91) or not (AOR = 1.75; 95 % CI = 1.39-2.20) in the last 14 days before clinical presentation.

Results for the analysis on each of the five most common MVI were very similar to those for all FRI analysis (Figs. 2 and 3). There was significantly higher risk of infection for all of the five most common MVI in stay-out personnel with ill household members compared with those who did not (Fig. 3). Regardless of whether they had ill household members or not, stay-in personnel with no ill bunkmates were not at significantly increased risk for any of the five most common MVI compared to stay-out personnel (with no ill household members). Stay-in personnel with ill bunkmates but without ill household members had significantly increased risk of all the MVI except Adenovirus E (AOR = 1.86; 95 % CI = 0.83-4.13), where there was a non-significant increase in risk; having ill household members further increased the risk for all five MVI over the reference category of stay-out personnel with no ill household members.

## Discussion

FRI may result in the loss of operational and training efficiency in the military setting [6, 13, 19, 26, 29, 37, 56]. Identification of risk factors can provide guidance to prevention and control measures to minimize morbidity and loss in operational efficiency. Although risk factors of FRI have been investigated in other work [29–32, 34, 35, 37], they may not be generalizable to all settings of interest. Furthermore, thus far, limited studies [52] had simultaneously document the risk of FRI due to a range of specific pathogens. In this study, we had shown that the five most common viral pathogens within our military environment was strongly associated with contact history, and had fairly similar trend of the FRI risk factors identified.

### Risk factors of FRI and mono-viral infections

Increasing age, recruit camp, and smokers were demographic risk factors for FRI. Increasing age was also reported as a risk factor for ARI in US military personnel in overseas deployments [37]. Additional analyses showed that the risk was higher with increasing age for all the five MVI in this study, but only significantly so for coxsackie/echovirus. In contrast, increasing age was previously reported to be a protective factor for seroconversion against influenza A(H1N1) pdm09 in the local military during the initial wave of infections from June to October 2009 [47]. These discrepant findings may be due to the changing age distribution of susceptible population towards influenza A(H1N1)pdm09 infections [57, 58], which might have shifted to involve more older individuals over the study period presented here (up to October 2012). In addition, this may be due to the increased in herd immunity effects among the new young cohorts of conscripts, where vaccine (initially as a monovalent formulation, and then later as part of the post-pandemic trivalent inactivated vaccine) was administered to all military recruits since November 2009 [23, 24]. Moreover, the lack or waning immunity against influenza A(H1N1)pdm09 in the older cohorts may have attributed to this trend, even though the individual level effects of vaccination against influenza A(H1N1)pdm09 (which was found to be significantly protective) was accounted for in the multivariate model.

Personnel in the recruit camp were at higher risk of FRI as well as all the five most common MVI, particularly adenovirus E infection. This is likely due to the higher contact exposure rate in semi-closed environments, and increased stressors [5, 7, 19, 21, 22, 29, 31, 38, 46]. Alternatively, it could be due to the fact that personnel in non-recruit camps are already protected due to the adaptive immune response developed from the previous infections in recruit camp, where recruits usually only stay on a short term basis, before posted to non-recruit camp.

Smoking has been shown to increase risk of upper respiratory infection among recruits [36], Hajj medical mission personnel [59], infants and children exposed to parental smoking [60]. Hence, it is not surprising to observe smoking as a risk factor of FRI in our study. There are some studies that had shown that cigarette smoking impairs oral and respiratory tract immunity [61–63]. This may have predispose smokers to a higher chance of viral infection resulting in FRI. However, further study is warranted to investigate mechanism behind this observation.

Malay ethnicity was positively associated with both influenza-A(H1N1)pdm09 and coxsackie/ echovirus mono-infections. We had previously also found Malays in the community to be at higher risk of influenza A(H1N1)pdm09 infection [64]. However, a previous study in the SAF found that Malays conscripts actually had significantly higher score in hygiene practices and knowledge towards pandemic influenza as compared to Chinese and Indians [65]. Hence, there may be a potential genetic basis for the higher risk of infection in Malays as compared to Chinese and Indians, given differences in genetic backgrounds of the HLA class 1 region which have been shown to result in weaker immune response against pathogen antigens [66]. Nevertheless, other unmeasured sociocultural and behavioural factors might explain these observations, and further studies are needed to confirm these observations and to understand the basis for the association.

### Effect of influenza vaccine

The protective effects of the influenza vaccine was largely in line with expectations, with the pre-pdm TIV protecting against influenza B but not against influenza A(H1N1)pdm09, the pdm-A(H1N1)V protecting against influenza A(H1N1)pdm09 but not influenza B, and the post-pdm TIV protecting against both pdm-A(H1N1)V subtypes, as observed in our previous study [24]. However, there were also some unexpected findings. These includes a potential protective effect (AOR = 0.61; 95 % CI = 0.43-0.86) of the pdm-A(H1N1)V against rhinovirus, and an increased risk (AOR = 49.51; 95 % CI = 32.91-74.48) of adenovirus E infection with the post-pdm TIV. These findings may have been due to non-specific interactions and interference between respiratory viruses which have been suggested by others [38], but could also have been due to the periodic nature of respiratory virus outbreaks. In particular, the post-pdm TIV period included a period of heightened adenovirus E activity (see Fig. 1) which might have been unrelated to changes in the vaccination policy, but which we could not adjust for due to co-linearity between the timing of these adenovirus E outbreaks and the phased roll-out of the influenza vaccine formulations. These unexpected findings would still require more scientific and epidemiological evidence for further conclusion.

### Contact history as risk factors

Travelling overseas in the last 14 days before clinical presentation was associated with a significantly increased risk for adenovirus E infection. We were not able to distinguish these as either military or personal overseas trips, but a previous outbreak of B2 human adenovirus E11a strain in a military camp in Singapore was also reported to be highly similar to other Asian strains involved in outbreaks, suggesting a potential import of this strain from the neighbouring regions [19]. As such, implementation of adenovirus vaccination may be useful to prevent sudden surge of cases with adenovirus E outbreak, given the high incidence of adenovirus infection in South-East Asia [19, 67].

One key finding was the relatively lower risk of FRI and the five most common MVI for stay-in personnel as compared with stay-out personnel. At least for influenza B and A(H1N1)pdm09, this could be due to the lower proportion of members in the households and the community who had the seasonal influenza vaccination [47], as compared to the camps where vaccination programme was implemented for all military personnel since the end of 2009 [23]. As such, this may have resulted in a smaller pool of susceptible individuals and a larger herd immunity effects in camps as compared to within the community. The other explanation maybe that stay-in personnel have less exposure to younger household members, which was previously found to have a significant risk for seroconversion to influenza A(H1N1)pdm09, and the risk was accentuated if the household member had FRI [47]. This also concurs with our findings on the effect of exposure to ill household members and bunkmates, and the effects are influenced by the domiciliary status of the soldier.

For the five most common MVI, an ill household member was a major risk factor for stay-out personnel. Moreover, the increase in risk for stay-in personnel from having ill household members was not as marked and mostly not significant. However, stay-in personnel with an ill bunkmate had a substantial increase in risk of infection. While our current study design does not allow us to attribute the cause of infection to contact with these ill household members or bunkmates, our findings do suggest that some of the transmission of these pathogens is mediated through close contacts, and support the use of preventive measures for FRI aimed at reducing transmission from ill household members and bunkmates. This could be in the form of issuing advisories to emphasize hygiene during outbreaks, and identifying and isolating ill personnel early to break the transmission of FRI. Moreover, this finding also has potential applications in surveillance. We had previously reported on how it would be difficult for syndromic surveillance systems to detect outbreaks in larger military units given the high baseline rates of respiratory illness [68]. Given that the relevance of ill bunkmates is consistent for the predominant viral agents of FRI, outbreak detection methods could instead focus on clusters of illness in those who share the same quarters, or are from the same military subunit as a reasonable proxy. We believe such an approach to syndromic surveillance deserves a prospective validation study where such clusters of illness are systematically sampled.

### Limitations

There are several limitations to this study. First, there was the influenza A (H1N1)pdm09 pandemic in June to September 2009 during the early part of the study period, where the force of infection for influenza A (H1N1)pdm09 is likely to be higher than usual. However, the pandemic spread was well-contained with prompt protective and preventive measures such as vaccination (Fig. 1), enhanced respiratory hygiene measures, isolation, quarantine, “ring prophylaxis” with oseltamivir during this period. As such, these measure are also likely to limit the risk of transmission of other circulating respiratory viruses during this specific period compared to other periods in the study. Since different vaccines were used promptly and appropriately during the different study periods (Fig. 1), vaccine type was used as a surrogate to account for the potential bias due to the enhanced protective and preventive measures applied during the influenza A (H1N1)pdm09 pandemic. Nevertheless, this bias should be minimal because the controls were also recruited in the same period and camp as the cases. Second, hand washing behaviour, allergy and military rank were not evaluated as potential risk factors of FRI. This is because it was very challenging to accurately assess how frequent hand washing was performed by the soldiers. Moreover, the soldiers may also tend to report the expected favourable hand washing behaviour. Hence, the likelihood of recall bias and information bias are likely to be high and would make any form of interpretation challenging. Allergy was not evaluated because the symptoms are very broad to specifically define as an allergy, and there would be significant potential information bias as it is less likely to clinically diagnosed allergy as compared with asthma, diabetes, hypertension and heart disease. Furthermore, the aim of this study is not to study clinical signs and symptoms that are associated with FRI. We did not consider military rank due to fact that there is a significant number of cases that were recruited from the recruit camp, where the population is mainly made up of recruits as compared to non-recruit camp, where the population is mainly made up of higher ranks (Table 1; *p* < 0.010). As such, it would be biased to include military rank as one of the variables. Third, our data is limited to febrile presentations of viral respiratory infections and may not be applicable to milder acute respiratory infections. Fourth, there is a lack of clinical and laboratory confirmation of the ill household members and bunkmates, and such data are hence subjected to recall bias. Fifth, the prevalence for FRI and MVI is about 17 % and 1 % respectively. As such, OR values as proxies to RR would be similar for MVI, whereas the OR of the risk factors for FRI is likely an overestimation, to some extent, relative to RR. Sixth, the ResPlex I assay (Qiagen) was designed to also detect six bacterial respiratory pathogens. They were Mycoplasma pneumoniae, Chlamydophila pneumoniae, Legionella pneumomophila, Streptococcus pneumoniae, Neisseria meningitides and Haemophilus influenza 1, 2, 3. However, FRI subjects with bacterial causes were not excluded because one of the aims of the study is to determine the potential risk factors for FRI, regardless of any detected or undetected respiratory virus and/or bacteria. Lastly, this study involved predominantly young adult males in a military context, and hence, the results may not be generalizable to the overall population in the community, particularly for the contact risk factors. However, during the pandemic of influenza A(H1N1)pdm09, clustering of febrile respiratory illness by classroom contact among school children [69] and ill workplace contacts among healthcare workers were also observed [47]. Further studies in other settings such as nursing homes which collect contact history in a similar way should be attempted.

## Conclusion

Increasing age, smokers, recruit camp, stay-out personnel with ill household members and stay-in personnel with ill bunkmates were independent risk factors of FRI in a semi-closed military setting. Early identification and isolation of ill bunkmates may be effective to prevent and to reduce further transmission in camp. Public health campaigns and policy should take these risk factors into consideration to increase the effectiveness of interventions to reduce FRI in the military environment.

## Abbreviations

FRI: Febrile respiratory illness
AOR: Adjusted odds ratio
CI: Confidence interval
SAF: Singapore Armed Forces
MVI: Mono-viral infection
